# [3,3′-Dihy­droxy-3,3′-bis­(pyridin-3-yl-κ*N*)-1,1′-(pyridine-2,6-di­yl)dipropan-1-one](nitrato-κ^2^ *O*,*O*′)silver(I)

**DOI:** 10.1107/S1600536811020472

**Published:** 2011-06-11

**Authors:** Jian-Yu Dong, Tian-Pa You

**Affiliations:** a Department of Chemistry, University of Science and Technology of China, Hefei, Anhui 230026, People’s Republic of China

## Abstract

In the title compound, a new macrocyclic metal complex, [Ag(NO_3_)(C_21_H_15_N_3_O_4_)], all non-H atoms are in a close-to-planar geometry (except for the nitrate anion), with a maximum out-of-plane deviation of 0.327 (6) Å for a pyridine C atom. The dihedral angle between the least-squares plane through the [3,3′-dihy­droxy-3,3′-bis­(pyridin-3-yl)-1,1′-(pyridine-2,6-di­yl)dipropan-1-one]silver(I) fragment and the nitrate anion is 31.29 (13)°. The mol­ecular structure is stabilized by several inter- and intra­molecular O—H⋯O and C—H⋯O hydrogen bonds. The Ag^I^ atom is coordinated by two pyridine N atoms and two O atoms of the nitrate anion in a geometry intermediate between tetrahedral and square-planar.

## Related literature

For general background, see: Zou *et al.* (2011 ▶), and references therein. For the synthesis of the ligand, see: Xi *et al.* (2008 ▶).
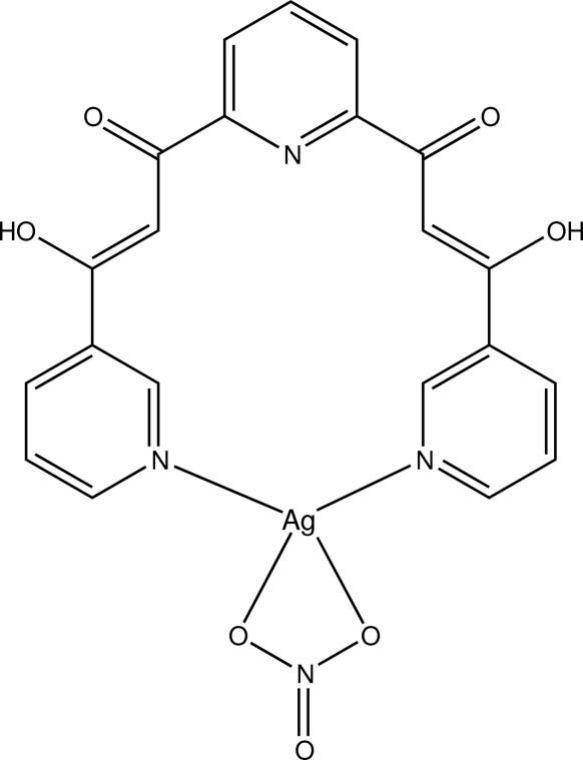


## Experimental

### 

#### Crystal data

[Ag(NO_3_)(C_21_H_15_N_3_O_4_)]
*M* *_r_* = 543.24
Triclinic, 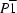

*a* = 6.5972 (14) Å
*b* = 12.572 (3) Å
*c* = 12.731 (3) Å
α = 101.256 (3)°
β = 101.610 (3)°
γ = 96.454 (3)°
*V* = 1001.7 (4) Å^3^
*Z* = 2
Mo *K*α radiation
μ = 1.06 mm^−1^
*T* = 298 K
0.33 × 0.27 × 0.19 mm

#### Data collection

Bruker SMART 1K CCD area-detector diffractometer
Absorption correction: multi-scan (*SADABS*; Sheldrick, 1996 ▶) *T*_min_ = 0.721, *T*_max_ = 0.824
5286 measured reflections
3478 independent reflections
2396 reflections with *I* > 2σ(*I*)
*R*_int_ = 0.021

#### Refinement

*R*[*F* ^2^ > 2σ(*F* ^2^)] = 0.041
*wR*(*F* ^2^) = 0.103
*S* = 1.06
3478 reflections
298 parameters
H-atom parameters constrained
Δρ_max_ = 0.49 e Å^−3^
Δρ_min_ = −0.41 e Å^−3^

### 

Data collection: *SMART* (Bruker, 2005 ▶); cell refinement: *SAINT* (Bruker, 2005 ▶); data reduction: *SAINT*; program(s) used to solve structure: *SHELXTL* (Sheldrick, 2008 ▶); program(s) used to refine structure: *SHELXTL*; molecular graphics: *SHELXTL*; software used to prepare material for publication: *SHELXTL*.

## Supplementary Material

Crystal structure: contains datablock(s) global, I. DOI: 10.1107/S1600536811020472/ff2013sup1.cif

Structure factors: contains datablock(s) I. DOI: 10.1107/S1600536811020472/ff2013Isup2.hkl

Additional supplementary materials:  crystallographic information; 3D view; checkCIF report

## Figures and Tables

**Table 1:** Hydrogen-bond geometry (Å, °)

*D*—H⋯*A*	*D*—H	H⋯*A*	*D*⋯*A*	*D*—H⋯*A*
C20—H20⋯O7^i^	0.93	2.53	3.386 (6)	154
C12—H12⋯O7^ii^	0.93	2.51	3.143 (5)	126
C9—H9⋯O6^iii^	0.93	2.56	3.289 (6)	136
C4—H4*A*⋯O1^iv^	0.93	2.33	3.205 (5)	157
O4—H4⋯O3	0.82	1.85	2.572 (4)	147
O2—H2⋯O1	0.82	1.80	2.531 (4)	147

Symmetry codes: (i) 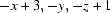; (ii) 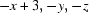; (iii) 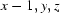; (iv) 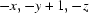.

**Fig. 1.**
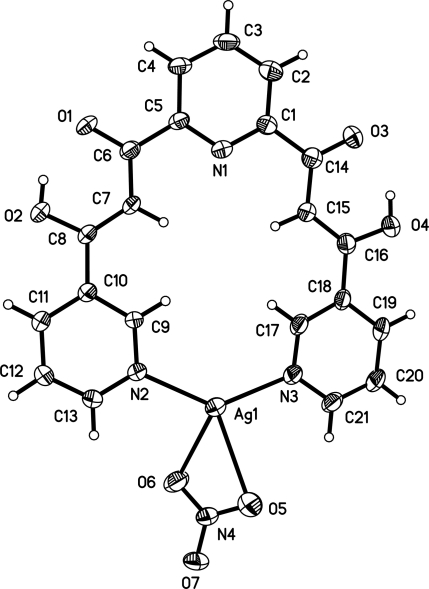
The molecular structure of the title compound, showing 30% probability displacement ellipsoids.

## supplementary crystallographic information

### Comment

The bridging pyridyl compounds are useful ligands to construct metal-cyclic complexes. We here report the synthesis and crystal structure of a novel macrocyclic silver complex containing a bridging pyridyl compound.

The sixteen-component chelate ring is not seen very often. The 16-membered chelate ring composed of atoms Ag1, N2, C9, C10, C8, C7, C6, C5, N1, C1, C14, C15, C6, C18, C17 and N3 has a nearly planar conformation [maximum deviation = 0.117 (5) Å for atom C9]. The molecular structure is stabilized by several intermolecular and intramolecular hydrogen bonds (Table 1).

Bond lengths and angles in the title molecule (Fig. 1) are within normal ranges.

### Experimental

3,3'-(pyridine-2,6-diyl)bis(1-(pyridin-3-yl)propane-1,3-dione) was synthesized as the reference method (Xi *et al.*, 2008). The title compound was prepared as the following method: 3,3'-(pyridine-2,6-diyl)bis(1-(pyridin-3-yl)propane-1,3-dione) (0.373 g, 1.0 mmol) and AgNO_3_ (0.168 g, 1.0 mmol) in 5 ml of DMF were stirred at room temperature for 12 h. The mixture was filtered and afforded the colourless solution. Colourless single crystals suitable for X-ray diffraction were obtained by slow diffusion of diethyl ether into the DMF solution.

### Refinement

H atoms were placed in calculated positions with C—H = 0.93–0.99 Å, and refined in riding mode with *U*_iso_(H) = 1.2*U*_eq_(C).

### Crystal data

**Table:**

[Ag(NO_3_)(C_21_H_15_N_3_O_4_)]	*Z* = 2
*M**_r_* = 543.24	*F*(000) = 544
Triclinic, *P*1	*D*_x_ = 1.801 Mg m^−^^3^
*a* = 6.5972 (14) Å	Mo *K*α radiation, λ = 0.71073 Å
*b* = 12.572 (3) Å	Cell parameters from 2396 reflections
*c* = 12.731 (3) Å	θ = 2.1–25°
α = 101.256 (3)°	µ = 1.06 mm^−^^1^
β = 101.610 (3)°	*T* = 298 K
γ = 96.454 (3)°	Block, colourless
*V* = 1001.7 (4) Å^3^	0.33 × 0.27 × 0.19 mm

### Data collection

**Table:**

Bruker SMART 1K CCD area-detector diffractometer	3478 independent reflections
Radiation source: fine-focus sealed tube	2396 reflections with *I* > 2σ(*I*)
graphite	*R*_int_ = 0.021
φ and ω scans	θ_max_ = 25.0°, θ_min_ = 2.1°
Absorption correction: multi-scan (*SADABS*; Sheldrick, 1996)	*h* = −7→7
*T*_min_ = 0.721, *T*_max_ = 0.824	*k* = −14→12
5286 measured reflections	*l* = −15→14

### Refinement

**Table:**

Refinement on *F*^2^	Primary atom site location: structure-invariant direct methods
Least-squares matrix: full	Secondary atom site location: difference Fourier map
*R*[*F*^2^ > 2σ(*F*^2^)] = 0.041	Hydrogen site location: inferred from neighbouring sites
*wR*(*F*^2^) = 0.103	H-atom parameters constrained
*S* = 1.06	*w* = 1/[σ^2^(*F*_o_^2^) + (0.045*P*)^2^ + 0.3559*P*] where *P* = (*F*_o_^2^ + 2*F*_c_^2^)/3
3478 reflections	(Δ/σ)_max_ = 0.001
298 parameters	Δρ_max_ = 0.49 e Å^−^^3^
0 restraints	Δρ_min_ = −0.41 e Å^−^^3^

### Special details

**Table:**

Geometry. All e.s.d.'s (except the e.s.d. in the dihedral angle between two l.s. planes) are estimated using the full covariance matrix. The cell e.s.d.'s are taken into account individually in the estimation of e.s.d.'s in distances, angles and torsion angles; correlations between e.s.d.'s in cell parameters are only used when they are defined by crystal symmetry. An approximate (isotropic) treatment of cell e.s.d.'s is used for estimating e.s.d.'s involving l.s. planes.
Refinement. Refinement of *F*^2^ against ALL reflections. The weighted *R*-factor *wR* and goodness of fit *S* are based on *F*^2^, conventional *R*-factors *R* are based on *F*, with *F* set to zero for negative *F*^2^. The threshold expression of *F*^2^ > σ(*F*^2^) is used only for calculating *R*-factors(gt) *etc*. and is not relevant to the choice of reflections for refinement. *R*-factors based on *F*^2^ are statistically about twice as large as those based on *F*, and *R*-factors based on ALL data will be even larger.

### Fractional atomic coordinates and isotropic or equivalent isotropic displacement parameters (Å^2^)

**Table:**

	*x*	*y*	*z*	*U*_iso_*/*U*_eq_	
Ag1	1.13449 (6)	0.10254 (4)	0.22826 (3)	0.0692 (2)	
N1	0.3450 (5)	0.3863 (3)	0.2614 (3)	0.0450 (9)	
N2	0.9937 (5)	0.1445 (3)	0.0679 (3)	0.0488 (9)	
N3	1.0707 (5)	0.1360 (3)	0.3959 (3)	0.0521 (10)	
N4	1.5330 (6)	0.0178 (3)	0.2232 (3)	0.0557 (10)	
O1	0.2448 (5)	0.4261 (3)	−0.0112 (2)	0.0634 (9)	
O2	0.4881 (5)	0.3291 (3)	−0.1132 (2)	0.0656 (10)	
H2	0.3933	0.3645	−0.1045	0.098*	
O3	0.3401 (5)	0.3916 (3)	0.5397 (3)	0.0602 (9)	
O4	0.6402 (5)	0.2954 (3)	0.6191 (2)	0.0620 (9)	
H4	0.5402	0.3286	0.6193	0.093*	
O5	1.4447 (6)	−0.0098 (3)	0.2916 (3)	0.0869 (12)	
O6	1.4707 (6)	0.0916 (4)	0.1789 (4)	0.0944 (14)	
O7	1.6850 (5)	−0.0217 (3)	0.2017 (3)	0.0801 (11)	
C1	0.2705 (7)	0.4022 (4)	0.3526 (4)	0.0477 (11)	
C2	0.0891 (7)	0.4478 (4)	0.3574 (4)	0.0577 (13)	
H2A	0.0374	0.4561	0.4208	0.069*	
C3	−0.0116 (7)	0.4803 (4)	0.2665 (4)	0.0644 (14)	
H3	−0.1324	0.5116	0.2683	0.077*	
C4	0.0641 (7)	0.4669 (4)	0.1741 (4)	0.0562 (12)	
H4A	−0.0014	0.4903	0.1129	0.067*	
C5	0.2431 (6)	0.4170 (4)	0.1730 (3)	0.0445 (11)	
C6	0.3270 (6)	0.3927 (4)	0.0730 (4)	0.0472 (11)	
C7	0.4910 (6)	0.3302 (4)	0.0706 (3)	0.0473 (11)	
H7	0.5504	0.3085	0.1341	0.057*	
C8	0.5655 (6)	0.3005 (4)	−0.0214 (3)	0.0457 (11)	
C9	0.8255 (7)	0.1952 (4)	0.0628 (3)	0.0489 (12)	
H9	0.7632	0.2034	0.1226	0.059*	
C10	0.7390 (6)	0.2361 (4)	−0.0262 (3)	0.0425 (10)	
C11	0.8261 (7)	0.2181 (4)	−0.1166 (4)	0.0581 (13)	
H11	0.7708	0.2424	−0.1792	0.070*	
C12	0.9963 (8)	0.1633 (4)	−0.1130 (4)	0.0618 (14)	
H12	1.0570	0.1503	−0.1733	0.074*	
C13	1.0752 (7)	0.1284 (4)	−0.0200 (4)	0.0491 (11)	
H13	1.1906	0.0920	−0.0183	0.059*	
C14	0.3917 (7)	0.3682 (4)	0.4490 (4)	0.0502 (11)	
C15	0.5630 (7)	0.3115 (4)	0.4354 (4)	0.0528 (12)	
H15	0.5966	0.2973	0.3670	0.063*	
C16	0.6790 (7)	0.2776 (4)	0.5204 (4)	0.0489 (11)	
C17	0.9098 (7)	0.1867 (4)	0.4105 (4)	0.0514 (12)	
H17	0.8256	0.2017	0.3489	0.062*	
C18	0.8581 (7)	0.2187 (4)	0.5100 (3)	0.0472 (11)	
C19	0.9853 (8)	0.1955 (5)	0.6011 (4)	0.0709 (16)	
H19	0.9584	0.2159	0.6706	0.085*	
C20	1.1518 (8)	0.1418 (5)	0.5870 (4)	0.0694 (15)	
H20	1.2377	0.1252	0.6471	0.083*	
C21	1.1907 (7)	0.1131 (4)	0.4856 (4)	0.0576 (13)	
H21	1.3034	0.0765	0.4774	0.069*	

### Atomic displacement parameters (Å^2^)

**Table:**

	*U*^11^	*U*^22^	*U*^33^	*U*^12^	*U*^13^	*U*^23^
Ag1	0.0654 (3)	0.1031 (4)	0.0590 (3)	0.0484 (2)	0.02162 (19)	0.0377 (2)
N1	0.041 (2)	0.044 (2)	0.051 (2)	0.0124 (17)	0.0075 (17)	0.0128 (18)
N2	0.048 (2)	0.061 (3)	0.048 (2)	0.0243 (19)	0.0163 (17)	0.0234 (19)
N3	0.044 (2)	0.069 (3)	0.051 (2)	0.018 (2)	0.0092 (17)	0.028 (2)
N4	0.047 (2)	0.065 (3)	0.059 (3)	0.024 (2)	0.0098 (19)	0.019 (2)
O1	0.067 (2)	0.082 (3)	0.054 (2)	0.0439 (19)	0.0091 (16)	0.0328 (18)
O2	0.073 (2)	0.091 (3)	0.0476 (19)	0.046 (2)	0.0133 (16)	0.0331 (18)
O3	0.069 (2)	0.070 (2)	0.0487 (19)	0.0200 (18)	0.0253 (16)	0.0144 (17)
O4	0.065 (2)	0.087 (3)	0.0440 (19)	0.0267 (19)	0.0216 (15)	0.0224 (17)
O5	0.089 (3)	0.103 (3)	0.095 (3)	0.037 (2)	0.047 (2)	0.044 (3)
O6	0.081 (3)	0.125 (4)	0.122 (3)	0.060 (3)	0.048 (2)	0.084 (3)
O7	0.069 (2)	0.112 (3)	0.076 (2)	0.058 (2)	0.0250 (19)	0.026 (2)
C1	0.050 (3)	0.047 (3)	0.047 (3)	0.013 (2)	0.014 (2)	0.008 (2)
C2	0.055 (3)	0.057 (3)	0.062 (3)	0.016 (3)	0.020 (2)	0.005 (3)
C3	0.048 (3)	0.075 (4)	0.071 (3)	0.031 (3)	0.013 (2)	0.009 (3)
C4	0.044 (3)	0.067 (3)	0.061 (3)	0.026 (2)	0.011 (2)	0.016 (3)
C5	0.041 (2)	0.045 (3)	0.048 (3)	0.015 (2)	0.005 (2)	0.014 (2)
C6	0.043 (2)	0.050 (3)	0.052 (3)	0.014 (2)	0.008 (2)	0.020 (2)
C7	0.042 (2)	0.064 (3)	0.042 (2)	0.021 (2)	0.0058 (19)	0.026 (2)
C8	0.045 (2)	0.053 (3)	0.044 (3)	0.017 (2)	0.006 (2)	0.022 (2)
C9	0.053 (3)	0.063 (3)	0.047 (3)	0.031 (2)	0.021 (2)	0.025 (2)
C10	0.042 (2)	0.046 (3)	0.042 (2)	0.011 (2)	0.0092 (19)	0.016 (2)
C11	0.064 (3)	0.078 (4)	0.048 (3)	0.033 (3)	0.019 (2)	0.031 (3)
C12	0.068 (3)	0.083 (4)	0.051 (3)	0.035 (3)	0.029 (2)	0.027 (3)
C13	0.044 (2)	0.061 (3)	0.049 (3)	0.019 (2)	0.016 (2)	0.017 (2)
C14	0.051 (3)	0.048 (3)	0.052 (3)	0.006 (2)	0.013 (2)	0.012 (2)
C15	0.056 (3)	0.069 (3)	0.043 (3)	0.026 (3)	0.015 (2)	0.021 (2)
C16	0.051 (3)	0.054 (3)	0.044 (3)	0.008 (2)	0.014 (2)	0.014 (2)
C17	0.048 (3)	0.065 (3)	0.046 (3)	0.013 (2)	0.005 (2)	0.027 (2)
C18	0.046 (3)	0.060 (3)	0.041 (3)	0.009 (2)	0.009 (2)	0.024 (2)
C19	0.078 (4)	0.104 (5)	0.050 (3)	0.034 (3)	0.020 (3)	0.045 (3)
C20	0.066 (3)	0.098 (4)	0.056 (3)	0.030 (3)	0.006 (3)	0.043 (3)
C21	0.050 (3)	0.065 (3)	0.067 (3)	0.019 (2)	0.010 (2)	0.035 (3)

### Geometric parameters (Å, °)

**Table:**

Ag1—N3	2.228 (4)	C5—N1	1.338 (5)
Ag1—N2	2.253 (3)	C5—C6	1.481 (6)
Ag1—O6	2.435 (4)	C6—O1	1.269 (5)
Ag1—O5	2.695 (4)	C6—C7	1.408 (6)
N1—C1	1.337 (5)	C7—C8	1.362 (6)
N1—C5	1.338 (5)	C7—H7	0.9300
N2—C13	1.326 (5)	C8—C10	1.479 (6)
N2—C9	1.337 (5)	C9—C10	1.380 (5)
N3—C17	1.324 (5)	C9—H9	0.9300
N3—C21	1.352 (5)	C10—C11	1.379 (6)
N4—O5	1.223 (5)	C11—C12	1.379 (6)
N4—O7	1.223 (5)	C11—H11	0.9300
N4—O6	1.242 (5)	C12—C13	1.368 (6)
O1—C6	1.269 (5)	C12—H12	0.9300
O2—C8	1.312 (5)	C13—H13	0.9300
O2—H2	0.8200	C14—O3	1.258 (5)
O3—C14	1.258 (5)	C14—C15	1.424 (6)
O4—C16	1.313 (5)	C15—C16	1.364 (6)
O4—H4	0.8200	C15—H15	0.9300
C1—N1	1.337 (5)	C16—C18	1.478 (6)
C1—C2	1.391 (6)	C17—C18	1.375 (6)
C1—C14	1.489 (6)	C17—H17	0.9300
C2—C3	1.373 (7)	C18—C19	1.390 (6)
C2—H2A	0.9300	C19—C20	1.376 (7)
C3—C4	1.356 (6)	C19—H19	0.9300
C3—H3	0.9300	C20—C21	1.354 (7)
C4—C5	1.399 (6)	C20—H20	0.9300
C4—H4A	0.9300	C21—H21	0.9300
			
N3—Ag1—N2	134.26 (12)	C6—C7—H7	118.8
N3—Ag1—O6	128.04 (13)	O2—C8—C7	121.5 (4)
N2—Ag1—O6	92.00 (12)	O2—C8—C10	114.8 (4)
N3—Ag1—O5	91.79 (12)	C7—C8—C10	123.6 (4)
N2—Ag1—O5	133.95 (12)	N2—C9—C10	123.8 (4)
O6—Ag1—O5	48.39 (11)	N2—C9—H9	118.1
C1—N1—C5	118.7 (3)	C10—C9—H9	118.1
C13—N2—C9	117.6 (3)	C11—C10—C9	117.4 (4)
C13—N2—Ag1	124.0 (3)	C11—C10—C8	122.0 (4)
C9—N2—Ag1	118.2 (3)	C9—C10—C8	120.6 (4)
C17—N3—C21	117.3 (4)	C10—C11—C12	119.0 (4)
C17—N3—Ag1	117.9 (3)	C10—C11—H11	120.5
C21—N3—Ag1	124.7 (3)	C12—C11—H11	120.5
O5—N4—O7	121.7 (4)	C13—C12—C11	119.4 (4)
O5—N4—O6	118.2 (4)	C13—C12—H12	120.3
O7—N4—O6	120.0 (4)	C11—C12—H12	120.3
C8—O2—H2	109.5	N2—C13—C12	122.6 (4)
C16—O4—H4	109.5	N2—C13—H13	118.7
N4—O5—Ag1	89.7 (3)	C12—C13—H13	118.7
N4—O6—Ag1	101.9 (3)	O3—C14—C15	122.6 (4)
N1—C1—C2	122.0 (4)	O3—C14—C15	122.6 (4)
N1—C1—C2	122.0 (4)	O3—C14—C1	118.7 (4)
N1—C1—C14	116.5 (4)	O3—C14—C1	118.7 (4)
N1—C1—C14	116.5 (4)	C15—C14—C1	118.7 (4)
C2—C1—C14	121.5 (4)	C16—C15—C14	121.4 (4)
C3—C2—C1	118.5 (4)	C16—C15—H15	119.3
C3—C2—H2A	120.8	C14—C15—H15	119.3
C1—C2—H2A	120.8	O4—C16—C15	122.3 (4)
C4—C3—C2	120.3 (4)	O4—C16—C18	114.5 (4)
C4—C3—H3	119.8	C15—C16—C18	123.2 (4)
C2—C3—H3	119.8	N3—C17—C18	124.7 (4)
C3—C4—C5	118.5 (4)	N3—C17—H17	117.6
C3—C4—H4A	120.7	C18—C17—H17	117.6
C5—C4—H4A	120.7	C17—C18—C19	116.9 (4)
N1—C5—C4	121.9 (4)	C17—C18—C16	121.7 (4)
N1—C5—C4	121.9 (4)	C19—C18—C16	121.4 (4)
N1—C5—C6	116.3 (3)	C20—C19—C18	119.0 (5)
N1—C5—C6	116.3 (3)	C20—C19—H19	120.5
C4—C5—C6	121.7 (4)	C18—C19—H19	120.5
O1—C6—C7	120.8 (4)	C21—C20—C19	120.1 (4)
O1—C6—C7	120.8 (4)	C21—C20—H20	120.0
O1—C6—C5	119.1 (4)	C19—C20—H20	120.0
O1—C6—C5	119.1 (4)	N3—C21—C20	122.0 (4)
C7—C6—C5	120.1 (4)	N3—C21—H21	119.0
C8—C7—C6	122.3 (4)	C20—C21—H21	119.0
C8—C7—H7	118.8		

### Hydrogen-bond geometry (Å, °)

**Table:**

*D*—H···*A*	*D*—H	H···*A*	*D*···*A*	*D*—H···*A*
C20—H20···O7^i^	0.93	2.53	3.386 (6)	154.
C12—H12···O7^ii^	0.93	2.51	3.143 (5)	126.
C9—H9···O6^iii^	0.93	2.56	3.289 (6)	136.
C4—H4A···O1^iv^	0.93	2.33	3.205 (5)	157.
O4—H4···O3	0.82	1.85	2.572 (4)	147.
O2—H2···O1	0.82	1.80	2.531 (4)	147.

Symmetry codes: (i) −*x*+3, −*y*, −*z*+1; (ii) −*x*+3, −*y*, −*z*; (iii) *x*−1, *y*, *z*; (iv) −*x*, −*y*+1, −*z*.
